# Prognostic value of CD4+ T lymphopenia in non-small cell lung Cancer

**DOI:** 10.1186/s12885-022-09628-8

**Published:** 2022-05-11

**Authors:** Guillaume Eberst, Dewi Vernerey, Caroline Laheurte, Aurélia Meurisse, Vincent Kaulek, Laurie Cuche, Pascale Jacoulet, Hamadi Almotlak, Jean Lahourcade, Marie Gainet-Brun, Elizabeth Fabre, Françoise Le Pimpec-Barthes, Olivier Adotevi, Virginie Westeel

**Affiliations:** 1 Chest Disease Department, University Hospital, 3 Boulevard Fleming, 25030 Besançon, France; 2 Methodology and Quality of Life in Oncology Unit, University Hospital, Besançon, France; 3 Université de Bourgogne Franche-Comté, EFS BFC, INSERM, UMR1098, RIGHT, Besançon, France; 4 INSERM CIC-1431, Clinical Investigation Center in Biotherapy, Biomonitoring Platform, F-25000 Besançon, France; 5 Department of Medical Oncology, University Hospital, Besançon, France; 6 Department of Medical Oncology, Assistance Publique-Hôpitaux de Paris, Hôpital Européen Georges Pompidou, Paris, France; 7 Department of Thoracic surgery, Assistance Publique-Hôpitaux de Paris, Hôpital Européen Georges Pompidou, Paris, France

**Keywords:** CD4+ T lymphopenia, Non small cell lung cancer, Prognosis, CD8+ T lymphopenia - local/loco-regional NSCLC

## Abstract

**Background:**

There is a paucity of data regarding the prognostic influence of peripheral blood CD4+ T lymphopenia in non-small cell lung cancer (NSCLC). Therefore, we investigated the prognostic value of T lymphopenia in NSCLC.

**Materials:**

Treatment-naive patients with a pathological diagnosis of NSCLC, at clinical stage I to IV were included in the prospective TELOCAP1 study. Lymphocytes count was evaluated in peripheral blood by flow cytometry. CD4+ and CD8+ T lymphopenia were defined as an absolute count of < 500/μL and < 224/μL respectively. The prognostic value of T lymphopenia was analyzed in the whole population, in local/loco-regional (stage I-IIIB) and in advanced (stage IV) NSCLC disease, using the Kaplan-Meier method and Cox regression models for survival curves and multivariate analysis, respectively.

**Results:**

Between July 2010 and January 2014, 169 evaluable patients with clinical stage I to IV NSCLC were prospectively enrolled. The prevalence of CD4+ and CD8+ T lymphopenia was similar in the study population (around 29%). Patients with CD4+ T lymphopenia showed lower overall survival than those with CD4+ T lymphocytes count > 500/μL (median overall survival (OS) 16.1 versus 21.7 months, hazard ratio (HR): 1.616 [95% CI: 1.1–2.36], p = 0.012). This association with OS was especially marked in local/loco-regional NSCLC stages (median OS, 21.8 versus 72 months, respectively, HR: 1.88 [95% CI: 0.9–3.8], *p* = 0.035). Multivariate analysis confirmed the worse prognosis associated with CD4+ T lymphopenia in local/loco-regional NSCLC, but not in metastatic patients (HR 2.028 [95% CI = 1.065–3.817] *p* = 0.02). Restricted cubic spline analysis showed that patients with CD4+ T lymphocytes count ≤500/μL displayed a high risk of death regardless of NSCLC clinical stage. There was no obvious relationship between CD8+ T lymphopenia and clinical outcome.

**Conclusion:**

We identified CD4+ T lymphopenia as an independent prognostic factor in local/loco-regional stages of NSCLC and CD4+ T lymphopenia is also associated with a high risk of death, regardless of NSCLC clinical stage.

**Trial registration:**

EUDRACT: 2009-A00642–55.

## Highlights

This study investigates the unknown prognostic influence of peripheral blood CD4+ T lymphopenia in non-small cell lung cancer (NSCLC) with long-term follow-up

CD4+ but not CD8 + T lymphopenia was associated with poor survival in patients with localized NSCLC

The CD4+ T lymphopenia appears to be an independent prognostic factor for poor overall survival in local/loco-regional NSCLC.

CD4+ T lymphocyte count ≤500/μL in peripheral blood is associated with a high risk of death in NSCLC

## Introduction

Recent progress in the treatment of non-small cell lung cancer (NSCLC) includes the introduction of immunotherapy, especially immune checkpoint inhibitors [1]. Although the use of immunotherapy in NSCLC has shown promising results, there remains a lack of predictive biomarkers indicating treatment benefit from immunotherapy [2]. Therefore, a better understanding of patient immune response is needed.

Evidence supports the role of the immune system in lung cancer development [3]. Indeed, high levels of tumor-infiltrating lymphocytes (TILs) have been shown to be associated with longer survival, and a significant reduction in the risk of death in patients with NSCLC [4–7]. More recently, a report by Mascaux et al. suggested that lung carcinogenesis involves a dynamic co-evolution of tumor bronchial cells and a decrease in local immune response [8]. Because evaluation of TILs requires large lung cancer specimens, there are few data on TILs in patients with advanced NSCLC. A retrospective cohort of 159 stage III and IV NSCLC patients did not show any association between TILs and prognosis [9].

The anti-tumor immune response is provided by both adaptive and innate immunity, in which T lymphocytes play a central role. Although, CD8+ T lymphocytes (CD8 TL) have been considered to be the main protagonists, due to their cytotoxic activity on tumor cells, it is now clear that CD4+ T lymphocytes (CD4 TL) also play a critical role in orchestrating the antitumor immune response [10–12]. Tumor-reactive CD4 TL have been found to ensure recruitment of cytotoxic CD8 TL at the tumor site [13]. In cancer patients, a high density of tumor-infiltrating CD4 Th1 cells has been identified as a good prognostic marker in several human cancers, including lung cancer [14]. CD4 TL can also exert a direct antitumor activity that is independent of CD8 TL, by recruiting and activating innate immune cells, such as natural killer lymphocytes and macrophages [15].

The critical role of CD4 T cell in antitumor immunity is supported by the poor prognosis associated with CD4 T lymphopenia in several cancers [16, 17]. CD4+ T lymphopenia has been found to be an independent risk factor for early death and for febrile neutropenia in lymphoma, myeloma, sarcoma, breast carcinoma, digestive tract carcinoma and germ cell tumor [16]. Furthermore, CD4+ lymphopenia was associated with non-response to chemotherapy, suggesting the important role of CD4+ T cells in controlling tumor progression [17]. There is a paucity of data regarding the prognostic influence of peripheral blood CD4+ T lymphopenia in NSCLC. Therefore, in the present study, we analyzed the prognostic value on overall survival of CD4+ T lymphopenia in patients with stage I to IV NSCLC.

## Patients and methods

### Study population

Patients with a pathological diagnosis of NSCLC, clinical stage I to IV, were included between July 2010 and January 2014, at the University Hospital Jean Minjoz, in Besançon and the European Hospital Georges Pompidou (EHGP), Paris, France, in the prospective TELOCAP01 study (EUDRACT: 2009-A00642–55) [18, 19]. The TeloCap01 study is a prospective, multicenter, immune-monitoring study conducted in patients with stage I–IV NSCLC, whose primary objective was to evaluate the landscape of telomerase-specific CD4+ T-cell responses in patients with NSCLC. The prognostic value of CD4+ T lymphopenia was a secondary endpoint of the TELOCAP01 trial. Before any therapy, including surgery, we collected and isolated blood lymphocytes, serum and plasma, which were frozen until later analysis. Survival data were collected at 1 and 2 years after inclusion.

Patients who were HIV positive, those receiving corticosteroids treatment, those with immunosuppression or another cancer diagnosis (except for basal cell carcinoma of the skin and in situ carcinoma of the uterine cervix) were excluded from the TELOCAP01 study. Stage I, II, and III NSCLC were considered as local/loco-regional NSCLC (7th edition of the TNM [20]).

All patients provided written informed consent and the protocol was approved by the ethics committee CPP (Comité de Protection des Personnes) Ile de France IV on 07/09/2009. The Telocap01 study was conducted in accordance with the Declaration of Helsinki and Good Clinical Practice guidelines.

### Assessment of blood lymphocyte count

Fresh, peripheral blood samples were collected before any treatment. Phenotypic analysis of peripheral blood lymphocyte subsets and absolute numbers of T cells, CD4+ and CD8+, were determined by single platform flow cytometry using the TetraCXP® method, Flow-Count fluorospheres, and FC500® cytometer (Beckman Coulter, Villepinte, France) according to the manufacturer’s recommendations [21].

CD4+ T and CD8+ T lymphopenia were defined as absolute lymphocyte counts (ALC) < 500/μL and < 224/μL respectively, according to a previous study by D’Hautcourt JL et al. [22]. These thresholds correspond to the lower limit of normal at the laboratory of the French Blood Transfusion Centre (Etablissement Français du Sang) where the lymphocyte counts were performed by flow cytometry, as justified by the study of D’Hautcourt et al. [22].

### Statistics

Overall survival (OS) was defined as the time from the date of inclusion to the date of death from any cause, or the date of last follow-up, for patients who were alive at last contact. Patients last known to be alive were censored at the time of their last follow-up assessment. The endpoint date was July 2020. Continuous variables are presented as median (interquartile range) and categorical variables as number (percentage). The relationship between main patient characteristics and T cell counts was studied. Medians and percentages were compared using the Wilcoxon rank test and Chi-square test (or Fisher’s exact test, if appropriate), respectively. OS was estimated using the Kaplan-Meier method and described using median or rate at specific time points with 95% confidence intervals (95% CI). Follow-up was calculated using a reverse Kaplan-Meier estimation when feasible [18]. Cox proportional hazards regression was performed to estimate hazard ratios (HR) and 95% CIs for factors associated with OS. The association of baseline parameters with OS was first assessed using univariate Cox analyses and variables with a *p*-value ≤0.05 were entered into a final multivariate Cox regression model. When used continuously, the association between biological parameters and OS was investigated using the restricted cubic splines method with graphical evaluation. All analyses were performed using SAS version 9.3 (SAS Institute Inc., Cary, NC) and R software version 2.15.2 (R Development Core Team; http://www.r-project.org). A *p*-values ≤0.05 was considered statistically significant and all tests were two-sided.

## Results

### Influence of clinical parameters on peripheral T cell count in NSCLC

Between July 2010 and January 2014, 170 NSCLC patients with a clinical stage I to IV were enrolled for the prospective study TELOCAP1. Among them, T lymphocyte count could not be measured in one patient.

The characteristics of the 169 evaluable patients are detailed in Tables 1 and 2. Median age was 64.5 years (95% CI = 57.5–70.5 years). 1 hundred and ten patients (65%) were males. A total of 147 patients (87%) were current or former smokers. A majority of patients had an ECOG performance status of 0 or 1 (80%). The histological type was adenocarcinoma in 87 patients (62%). Molecular analyses were available in 79 patients and EGFR mutations were observed in 11 patients and KRAS mutations in 19 patients. The proportion of patients with local/loco-regional NSCLC was 51%. No patient received immunotherapy (Tables 1 and 2).

**Table 1:**
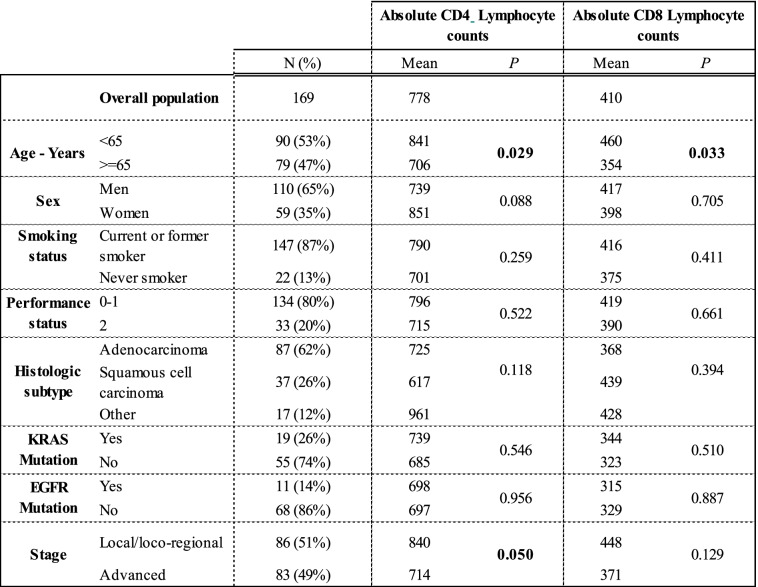
Absolute CD4 and CD8 lymphocyte counts according to patients’ characteristics. ^+^: large cell carcinoma, adenosquamous carcinoma and sarcomatoid carcinoma

**Table 2:** Patients’ characteristics according to CD4+ and CD8+ T lymphopenia

	Overall population (***N =*** 169)		Absolute TL CD4 count ≤ 500 (***N =*** 48)		Absolute TL CD4 count > 500 (***N =*** 121)		P	Absolute TL CD8 count ≤ 224 (***N =*** 50)		Absolute TL CD8 count > 224 (***N =*** 119)		P
		N		N		N			N		N	
**Clinical characteristics**												
**Age — years**	64.5 (57.5 - + 70.5)	169	65.5 (60.5–76.6)	48	63.7 (56.9–69.7)	121	0.0532	65.7 (61.9–72.1)	50	63.3 (56.8–70.5)	119	0.0925
**Patient male sex**	110 (65%)	169	33 (69%)	48	77 (64%)	121	0.5938	35 (70%)	50	75 (63%)	119	0.3853
**Smoking status**		169		48		121			50		119	
Current or former smoker	147 (87%)		42 (87%)		105 (87%)		1.000	45 (90%)		102 (85.7%)		
Never smoker	22 (13%)		6 (13%)		16 (13%)			5 (10%)		17 (14.3%)		0.4498
**Performance status OMS**		167		47		120			48		119	
0–1	134 (80%)		33 (70%)		101 (84%)		**0.0417**	39 (81.3%)		95 (79.3%)		
2	33 (20%)		14 (30%)		19 (16%)			9 (18.2%)		24 (20.2%)		0.8350
**Pathological characteristics**												
**Histologic subtype**		141		46		95			44		97	
Adenocarcinoma	87 (62%)		26 (57%)		61 (64%)		0.0813	29 (65.9%)		58 (59.8%)		
Squamous cell carcinoma	37 (26%)		17 (37%)		20 (21%)			11 (25%)		26 (26.8%)		
other	17 (12%)		3 (6%)		14 (15%)			4 (9.1%)		13 (13.4%)		0.7091
**KRAS mutation**	19 (26%)	74	5 (20%)	25	14 (29%)	49	0.5758	4 (15.4%)	26	15 (31.3%)	48	0.1358
**EGFR mutation**	11 (14%)	79	4 (15%)	26	7 (13%))	53	1.0000	4 (14.3%)	28	7 (13.7%)	51	1.000
**Stage**		169		48	20 (21%)	121			50		119	
Local/loco-regional	86 (51%)		21 (44%)		65 (54%)		0.2424	27 (54%)		59 (49.6%)		0.5998
Advanced	83 (49%)		27 (56%)		56 (46%)			23 (46%)		60 (50.4%)		

The T lymphocyte subset counts are summarized in **Table** **1**. In the overall cohort, the mean lymphocyte count was 778/μl and 410/μl for CD4 and CD8 subsets respectively. We observed that both CD4 and CD8 T cell counts declined with increasing age and NSCLC stage. As expected, the level of CD4 T lymphocytes was significantly lower in advanced stages compared to localized stages (710 versus 840 CD4/μL, *p* = 0.05). No obvious association was observed for the other main clinical parameters (**Table** **1**).

Next, to assess the prognostic value of T lymphocyte count (CD4 or CD8) in the whole cohort, we used thresholds to define CD4+ and CD8 + T lymphopenia (< 500/μL and < 224/μL) respectively [22]. In whole cohort, CD4+ T lymphopenia (< 500/μL) and CD8+ T lymphopenia (< 224/μL) were observed in 28.4 and 29.6% of patients respectively. CD4+ T lymphopenia was significantly more frequently observed in elderly patients (*p* = 0.053), and in patients with performance status ≥2 (*p* = 0.041). There was a trend towards an increased frequency of CD4+ T lymphopenia (56%) in metastatic patients versus 44% in patients with localized NSCLC (*p* = 0.24). No association was found between CD4+ or CD8+ count T cell counts and other variables such as gender, smoking, and histology (Table 2).

### CD4+ but not CD8 + T lymphopenia was associated with poor survival in NSCLC patients

**Fig. 1:**
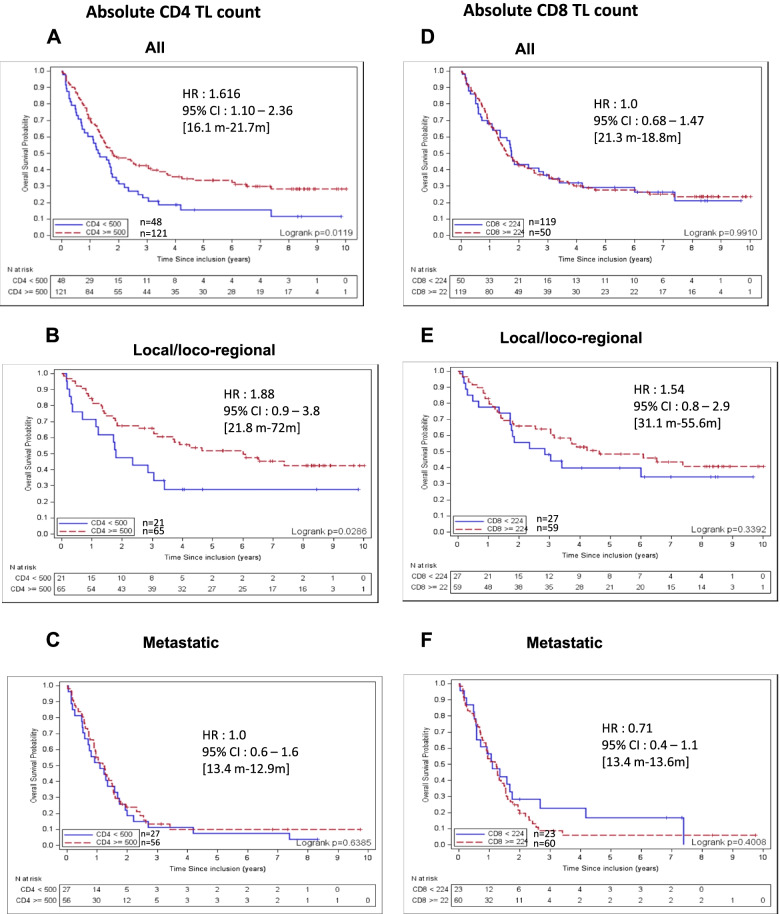
Overall Survival according to CD4 and CD8 T lymphopenia in NSCLC. Association between CD4 lymphopenia and overall survival in the whole cohort (A), local/locoregional (B) and metastatic disease (C). Association between CD8 lymphopenia and overall survival in the whole cohort (D), local/locoregional (E) and metastatic disease (F)

The estimated median OS was 20.4 months for the overall cohort, 44.8 months in local/loco-regional NSCLC and 13.4 months in metastatic patients. In the whole cohort, median OS was better for patients with CD4 TL counts > 500/μL compared with those who had CD4 + T lymphopenia (21.7 versus 16.1 months, respectively, HR: 1.616 [95% CI: 1.1–2.36], *p* = 0.012) (Fig. 1A). The favorable prognostic value of CD4 TL count > 500/μL was observed in patients with local/loco-regional disease (stage I to IIIB), but not in metastatic patients (median OS, 72 versus 21.8 months, respectively, HR: 1.88 [95% CI: 0.9–3.8], *p* = 0.0286) (Figs. 1B and C).

In contrast, no obvious association was visible between CD8+ T lymphopenia and survival (median OS 21.3 versus 18.8 months, HR: 1.0 [95% CI: 0.70–1.5] *p* = 0.991) (Fig. 1D). A trend towards a favorable prognostic value of CD8+ TL count > 224/μL was observed in patients with local/loco-regional disease but not in metastatic patients (median OS 55.6 versus 31.1 months, *p* = 0.339) (Figs.1E-F).

Univariate analyses showed that among the main patient characteristics, CD4 + T lymphopenia, total lymphocyte count, performance status ≥2 and advanced NSCLC were associated with worse OS (Table 3): CD4+ T lymphopenia (HR 1.61 [95% CI = 1.107; 2.358] *p* = 0.012); performance status ≥2 (HR 2.88 [95% CI = 1.88; 4.405] *p* < 0.0001); advanced stage (HR 3.03 [95% CI = 2.08; 4.43] p < 0.0001)); total lymphocyte count < 1000/μL (HR 1.65 [IC 95% = 1.06; 2.56] *p* = 0.0262). In contrast, no association was found between the absolute CD8 T cell count and OS (Table 3). Colinearity was observed between immunological parameters. Only CD4+ T lymphopenia was included in the multivariate analysis because among the immunological parameters, this variable was most strongly associated with survival in univariate analysis.

**Table 3:** Univariate analysis for overall survival

	No. of patients	Nr of deaths	HR	95% CI	***P***
**Age — years**	169				
< 70	123	90	1		
≥ 70	46	32	1.074	0.717 to 1.608	0.7301
**Patient sex**	169				
Male	110	83	1		
Female	59	39	0.805	0.550 to 1.179	0.2651
**Smoking status**	169				
Current or former smoker	147	108	1		
Never smoker	22	14	0.813	0.465 to 1.419	0.4659
**Performance status OMS**	167				
0–1	134	91	1		
≥ 2	33	30	2.881	1.885 to 4.405	**<.0001**
**Histologic subtype**	141				
Adenocarcinoma	87	67	1		
Squamous cell carcinoma	37	31	1.221	0.797 to 1.871	
other	17	15	1.711	0.974 to 3.003	0.1549
**Stage**	169				
Local/Loco-regional (I-III)	86	49	1		
Advanced (IV)	83	73	3.039	2.083 to 4.433	**<.0001**
**CD8+ T Lymphocytes**	169				
≥ 224 /μl	119	86	1		
< 224 /μl	50	36	0.998	0.676 to 1.472	0.9903
**CD4+ T lymphocytes**	169				
≥ 500 /μl	121	81	1		
< 500 /μl	48	41	1.616	1.107 to 2.358	**0.0127**
**Total lymphocytes count**	169				
≥ 1000 /μl	129	73	1		
< 1000 /μl	40	28	1.65	1.06 to 2.56	**0.0262**

The multivariate analyses performed in whole cohort showed that performance status ≥2 (HR 2.191 [95% CI = 1.413; 3.396] *p* = 0.0005) and advanced stage (HR 2.558 [95% CI = 1.722; 3.802] *p* < 0.0001) were significantly correlated with poor survival, whereas a trend towards worse prognosis with CD4+ T lymphopenia was observed (HR 1.422 [95% CI = 0.971; 2.083] *p* = 0.0704) (Table 4**).** However, the multivariate analyses carried out in the subgroups of patients showed CD4+ T lymphopenia was significantly correlated with poor survival in local/loco-regional but not in metastatic patients (HR 2.028 [95% CI = 1.065; 3.817] *p* = 0.02) (Table 4).

**Table 4:** Multivariate analysis for overall survival

In All Patients					
	No. of patients	No. of deaths	HR	95% CI	***P***
**Performance status OMS**	167				
0–1	134	91	1		
≥ 2	33	30	2.191	1.413 to 3.396	**0.0005**
**Stage**	167				
Local/Loco-regional	84	48	1		
Advanced	83	73	2.558	1.722 to 3.802	**<.0001**
**CD4+ T lymphocytes**	167				
≥ 500 /μl	120	80	1		
< 500 /μl	47	41	1.422	0.971 to 2.083	**0.0704**
**In patients with local/loco-regional NSCLC**					
	**No. of patients**	**No. of deaths**	**HR**	**95% CI**	***P***
**Performance status OMS**	84				
0–1	78	43	1		
≥ 2	6	5	1.788	0.687 to 4.649	0.2335
**CD4+ T lymphocytes**	84				
≥ 500 /μl	64	33	1		
< 500 /μl	20	15	2.028	1.065 to 3.817	**0.0288**
**In Advanced NSCLC**					
	**No. of patients**	**No. of deaths**	**HR**	**95% CI**	***P***
**Performance status OMS**	83				
0–1	56	48	1		
≥ 2	27	25	2.270	1.354 to 3.806	**0.0019**
**CD4+ T lymphocytes**	83				
≥ 500 /μl	56	47	1		
< 500 /μl	27	26	1.198	0.737 to 1.946	**0.4668**

**Fig. 2:**
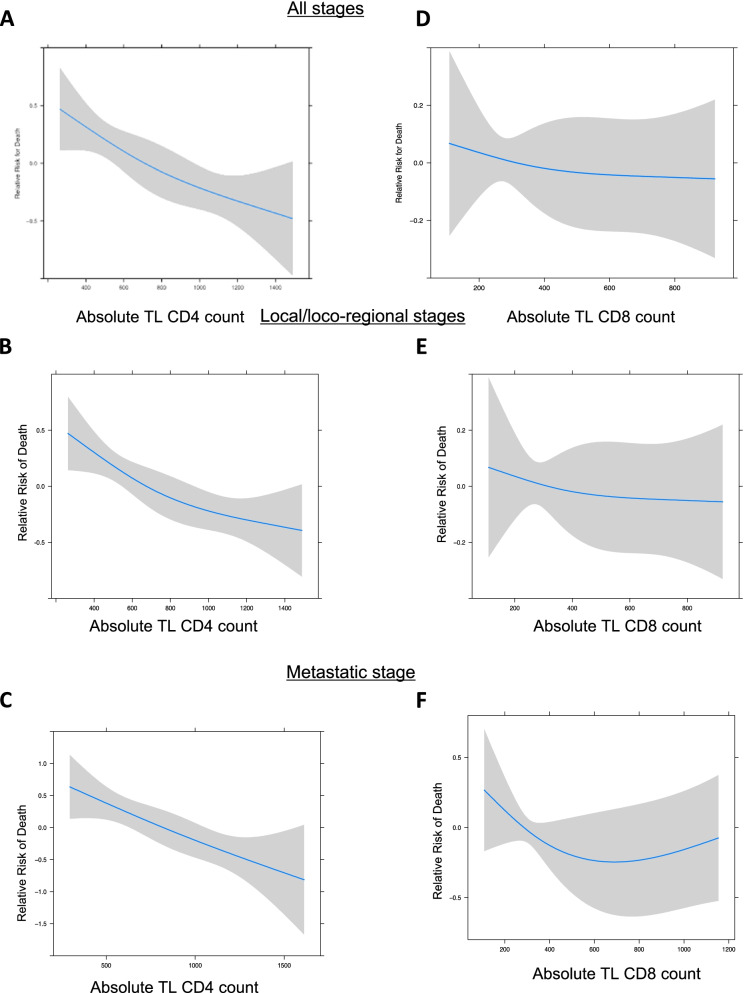
Risk of death according to lymphopenia in NSCLC stages. Restricted cubic splines modeling of hazard ratio (HR) for Overall Survival according to CD4 TL count (A to C) or CD8 TL count (D to F). The purple area around the blue line represents the 95% confidence interval

Next, the risk of death was analyzed using the restricted cubic spline (RCS) model, which characterizes a non-linear dose-response association between a continuous variable and an outcome. In the whole NSCLC cohort, the RCS model showed that patients with CD4 TL count ≤500/μL displayed a higher risk of death. Notably, the gradual risk observed in the whole cohort, local/loco-regional NSCLC and in metastatic stage suggested a linear relation between CD4 TL count and patient OS (Fig. 2A, B, C). In contrast, the RCS approach showed no linear relation between risk of death and CD8 + T cell count (Fig. 2D, E, F). Thus, low level of CD4 TL count in peripheral blood appears to be an independent risk factor for death in NSCLC.

## Discussion

In the present study, we identified the CD4+ T lymphopenia (count < 500/μL) as a poor prognostic factor associated with a high risk of death in NSCLC. However, no association between CD8+ TL lymphopenia and patients’ clinical outcome was found in this cohort. This suggests that in contrast to CD8 T cells, high levels of peripheral CD4 T cells play a protective role in NSCLC patients. This is the first prospective study addressing the prognostic value of CD4+ T lymphopenia in NSCLC with long-term follow-up.

Previous studies have reported that CD4 lymphopenia was associated with poor prognosis in cancer patients [16, 17, 23], but these studies did not include patients with lung cancer. Here, we found that metastatic patients were more frequently affected by CD4 lymphopenia than patients with localized disease. However, the results showed that CD4+ T lymphopenia was significantly associated with poor survival in local/loco-regional disease, but not in metastatic patients. An explanation may be related to functional impairment of CD4 T cells, regardless of their circulating levels. Indeed, chronic inflammation and the accumulation of immunosuppressive factors at the metastatic stage can lead to functional alterations in CD4 T lymphocytes, which play a crucial role in the immune surveillance of cancers [24–26].

The metastatic status is the result of an accumulation of co-founding clinical parameters (PS, site of metastasis …), as well as a chronic inflammatory, and immune-suppressive environment [8]. Interestingly, a CD4 TL count < 500/μL increases the risk of death in this cohort of both in localized and metastatic NSCLC. This is particularly relevant for the management of localized patients, to identify patients with poor outcomes after surgery and adapt the treatment, with adjuvant therapy or intensified follow-up. In line with our data, a high preoperative total lymphocyte count was shown to reduce 5-year OS and 5-year relapse-free survival rates in resected colorectal cancer [27]. Moreover, CD4 T lymphopenia was found to have prognostic value only in patients with localized disease in our study, so it would be interesting to explore factors involved in CD4 lymphopenia in this setting, such as immune senescence. Indeed, CD4 T lymphopenia was found significantly more frequently in older patients including in patients with localized disease (mean age around 70 years).

We found that only peripheral CD4 lymphopenia influences the prognosis of NSCLC patients. This finding could be related to the central role of CD4 T cell in antitumor immunity [10, 28]. Indeed, CD4 T cells, particularly the Th1 subset, control cell-mediated immunity against tumors and have a “helper” role towards antitumor CD8 T cells [12]. Consequently, tumor-infiltrating Th1 cells have been identified as a good prognostic marker in many human cancers [29, 30]. Furthermore, we recently reported that the presence of circulating tumor-specific CD4 Th1 was associated with better prognosis in lung cancer patients, notably in patients with localized disease [19]. Unlike peripheral CD4 TL, peripheral CD8 T lymphopenia did not appear to be related to patients’ survival. One possible explanation may be the fact that CD8 T cells predominantly act as effectors in the tumor microenvironment. Accordingly, evidence supports the prognostic value of CD8+ TILs reported in many cancers, including lung cancer [7, 14].

Although we found a statistical association with meaningful clinical implications for patient care, the low number of patients included in our study reduces the robustness of these results. Another limit of this study is the absence of information on treatments received by patients so that their link between CD4+ T lymphopenia could not be addressed. Thus, the use of CD4 lymphopenia in NSCLC as predictive biomarker deserves future confirmation even more during immunotherapy. Indeed, a large multicenter prospective immune monitoring study in NSCLC called TELOCAP2 (NCT NCT02846103) is under way, and will make it possible to address these limitations. These data also support the value of peripheral blood immune monitoring in NSCLC. Liquid biopsy (ctDNA), neutrophil to lymphocyte ratio (NLR), LDH, absolute lymphocyte count, MDSC, represent interesting potential blood biomarkers in lung cancer [31]. The poor prognosis associated with CD4 lymphopenia is widely documented in many cancers. Like other circulating biomarkers, such as the NLR, the assay is routinely done everywhere, with results being available quickly with standardized thresholds worldwide. For example, CD4 count is a good indicator of immune status, and is routinely used for the management of patients with HIV infection and other immunodeficiency disorders [32]. The deleterious effect of CD4 lymphopenia observed in patients with localized disease suggests an important role of these cells in cancer progression, as recently described in the study by Mascaux et al. [8]. In this regard, the role of peripheral CD4 TL has gained considerable interest for cancer immunotherapy in the last few years [33–36]. Hence, the critical role of peripheral CD4 T-cell populations but not CD8 TL for “real-time” blood-based monitoring has recently been highlighted in NSCLC patients treated with immune checkpoint inhibitors [37]. Finally, this study may suggest that CD4 TL count can guide the use of possible adjuvant therapy, according to the personalized risk for each patient.

## Conclusion

In the present study, we showed that CD4 TL count in the peripheral blood represents a promising prognostic factor for early stages of NSCLC. This is the first prospective study to address the prognostic value of CD4+ T lymphopenia in NSCLC with long-term follow-up of over 10 years.

## Data Availability

The data from this study are available from the corresponding author on reasonable written request. The data are not publicly available because them containing information that could compromise research participant privacy.

## Abbreviations

NSCLC: Non-Small Cell Lung Cancer
EGFR: Endothelial growth factor receptor
ALK: Anaplastic lymphoma kinase
PS: Performance status
TL: T lymphocyte
CTLA-4: Cytotoxic T-lymphocyte-associated protein 4
PD-1: Programmed cell death 1
PD-L1: Programmed cell death-ligand 1
TIL: Tumor-infiltrating lymphocyte
OS: Overall Survival
PFS: Progression Free Survival
HR: Hazard Ratio
